# Carbon storage in Ghanaian cocoa ecosystems

**DOI:** 10.1186/s13021-016-0045-x

**Published:** 2016-05-23

**Authors:** Askia M. Mohammed, James S. Robinson, David Midmore, Anne Verhoef

**Affiliations:** 1 CSIR-Savanna Agricultural Research Institute, Nyankpala, PO Box 52, Tamale, Ghana; 2 School of Archaeology, Geography and Environmental Science, University of Reading, Reading, UK

**Keywords:** Carbon stocks, Cocoa ecosystem, Shaded and unshaded cocoa systems

## Abstract

**Background:**

The recent inclusion of the cocoa sector as an option for carbon storage necessitates the need to quantify the C stocks in cocoa systems of Ghana.

**Results:**

Using farmers’ fields, the carbon (C) stocks in shaded and unshaded cocoa systems selected from the Eastern (ER) and Western (WR) regions of Ghana were measured. Total ecosystem C (biomass C + soil C to 60 cm depth) ranged from 81.8 to 153.9 Mg C/ha. The bulk (~89 %) of the systems’ C stock was stored in the soils. The total C stocks were higher in the WR (137.8 ± 8.6 Mg C/ha) than ER (95.7 ± 8.6 Mg C/ha).

**Conclusion:**

Based on the cocoa cultivation area of 1.45 million hectares, the cocoa sector in Ghana potentially could store 118.6–223.2 Gg C in cocoa systems with cocoa systems aged within 30 years regardless of shade management. Thus, the decision to include the cocoa sector in the national carbon accounting emissions budget of Ghana is warranted.

## Background

Cocoa is cultivated in the forest regions of Ghana where an estimated area of 1.45 million hectares of forest land has been displaced [1]. A substantial volume of literature is replete with evidence that the reductions in forest cover produced net sources of carbon dioxide (CO_2_), the main greenhouse gas of the atmosphere [2, 3]. According to the Intergovernmental Panel on Climate Change (IPCC), global C stocks in terrestrial biomass have decreased by 25 % over the past century [3, 4]. This corresponds to an annual decline of 1.1 Gt of the global carbon stocks in forest biomass [5]. Stern [2] note that deforestation alone is responsible for 18 % of the world’s greenhouse gas emissions.

Cocoa intensification for higher yields has led to a drastic reduction in shade tree density and, on many farms total elimination of the shade trees in cocoa ecosystems [6]. Essentially, cocoa expansion in Ghana has been closely linked to deforestation [7, 8]. One option to redress deforestation and create a carbon sink is to encourage the establishment of tree-crop farming or agroforestry systems [9–11]. Cocoa agroforestry is an age-old practice in the tropics [12]. Various recommendations have been made to farmers with regard to the number of non-cocoa trees to provide shade for cocoa during planting. However, the decision on how much shade is optimal often depends on the ecological system, social factors, biodiversity interests, ecological services and pod yields [7, 11].

With the recent inclusion of the cocoa sector in the national C emission accounting budgets of Ghana [13], the need to quantify the carbon sequestered in cocoa ecosystems is urgent. In addition to measuring the amounts of carbon stored in cocoa and shade tree biomass in the cocoa systems, the soil organic carbon content needs to be determined. Globally, the amount of C stored in soils is estimated to be 1.5–3 times more than in vegetation [9]. Thus, if Ghana is to include the C sequestered in the cocoa sector in its proposal for developing a national carbon accounting strategy, as outlined in its Readiness Plan Proposal [13], the C quantities stored both in the vegetation and the soils of the cocoa ecosystems must be included.

This paper evaluates the C storage in cocoa ecosystems from two regions of Ghana under two shade management systems and two cocoa stand age categories. It was hypothesised that; (a) the distribution of the total C stocks in the cocoa ecosystem differs between vegetation and soils, and (b) the C stocks differ between regions and shade management. The objectives were: (1) to quantify the total carbon stocks and distribution in the cocoa ecosystem, and (2) to assess the influence of shade management and the region of cocoa production on the C stocks.

## Results and discussion

### Selected properties of the soils under the cocoa ecosystems

The present study showed a range of 1.1–1.9 Mg/m^3^ as the bulk density of the soils under the cocoa ecosystems (Table 1). As expected, the bulk density increased with soil depth from the surface. The gravimetric moisture content of the soils under the cocoa ecosystems ranged from 12.6 to 17.9 % (w/w). The soil moisture only varied with soil depth with the topsoil, 0–20 cm, being the wettest (Table 1). The ranges of the particle size fractions were: clay, 6.6–13.6 %; sand, 49–53 %, and silt, 36–41 % (Table 1). The soils are characterised as having the texture of sandy silt throughout the 0–60 cm layer (Table 1).

**Table 1:** Grand mean ± standard error of selected properties of the soils in the cocoa ecosystems for region (n = 24), system (n = 24) and depth (n = 16)

Factor	Treatment	Bulk density (Mg/m^3^)	Clay	Sand	Silt	Moisture	C
			(%)				
Region	Eastern	1.5 ± 0.1	8.3 ± 0.6	51 ± 2	40 ± 1	14.7 ± 0.7	0.7 ± 0.1
	Western	1.6 ± 0.1	11.7 ± 0.6	52 ± 2	38 ± 1	14.7 ± 0.7	1.5 ± 0.1
System	Shaded	1.6 ± 0.1	10.5 ± 0.6	53 ± 2	36 ± 1	14.8 ± 0.7	1.0 ± 0.1
	Unshaded	1.5 ± 0.1	9.6 ± 0.6	49 ± 2	41 ± 1	14.7 ± 0.7	1.3 ± 0.1
Soil depth	0–20 cm	1.1 ± 0.1	6.6 ± 0.7	53 ± 2	41 ± 1	17.9 ± 0.8	2.0 ± 0.1
	20–40 cm	1.6 ± 0.1	9.9 ± 0.7	51 ± 2	39 ± 1	12.6 ± 0.8	0.8 ± 0.1
	40–60 cm	1.9 ± 0.1	13.6 ± 0.7	50 ± 2	37 ± 1	13.7 ± 0.8	0.6 ± 0.1

Age of farms appearing as covariate

### Biomass C concentrations

**Fig. 1:**
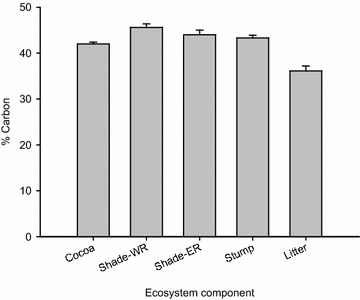
Percentage carbon in cocoa ecosystem components (*n* = 5). *Persea americana* and *Newbouldia laevis* are dominant shade trees in Western and Eastern regions, respectively. *Error bars* represent standard errors

The mean carbon concentrations in above-ground components for all of the ecosystems under evaluation are presented in Fig. 1. The measured litter carbon concentration of 36.1 ± 1.1 corroborates the value of 37 % C in forest litter by Smith and Heath [14] that is currently being used as a default C concentration for litter in agro-ecosystems by the Intergovernmental Panel on Climate Change [3]. Similarly, the current carbon concentration value of 42.0 ± 0.4 % for the cocoa trees is in agreement with 43.7 ± 2.1 % for cocoa carbon reported by Anglaaere [15].

With the exception of litter C (a proportion of which is lost through respiration as it decomposes), the other components had a narrow range of 42.0–45.6 % C, with cocoa trees having the least C and *Persea americana* (dominant shade species in the Western region) having the highest (Fig. 1). Although few studies on agroecosystem C stocks present direct measurements of carbon with the aid of a C-analyser [16, 17], several studies have used constant values ranging from 45 to 50 % as the proportion of C for all parts of tree biomass [18, 19]. The organic carbon levels in the shade trees in the current study are not markedly different from the constant 45 % C for forest species being used by other studies [20, 21].

### Soil organic carbon concentration

The soil total organic carbon concentrations differed significantly (P < 0.05) between regions, systems and soil depths (Table 1). Soil C concentration decreased with soil depth from the surface. Similar trends with depth have been noted by Cifuentes-Jara [22] and Dawoe [23]. The topsoil, 0–20 cm, contained approximately 58.8 % of the soil organic C in the 0–60 cm soil profile. This undoubtedly reflects the great mass of litter fall in cocoa ecosystems. In addition, the high C concentration in the topsoil is in accordance with the presence of 80–85 % mat of lateral roots of cocoa trees being predominantly found within the top 0–30 cm [23–25], although visible roots were excluded in sampling for the current study. The soil C concentration range of 0.6–2.0 % lies within the soil C concentration range of 0.4–2.6 %, reported by Dawoe [23] for 15 and 30 year old cocoa ecosystems in the Ashanti region, Ghana.

### Above-ground carbon stocks in cocoa ecosystems

The C contribution from different cocoa ecosystem components to the total above-ground biomass C varied among regions, system, and their interactions (Table 2). On a per hectare basis, the system’s biomass C components ranged as follows: cocoa trees, 11.8–16.9 Mg C/ha; shade trees, 10.2–16.4 Mg C/ha; litter, 1.9–2.9 Mg C/ha, and stumps, 0.01–0.24 Mg C/ha (Table 2). These results compared well with the ranges of C stocks reported for cocoa trees in the literature [26, 27]. Similarly, the C stocks in shade trees of the current study agreed with the estimates for those in agro-ecosystems researched by Kürsten and Burschel [28] and Polzot [29] (3–25 and 1.9–31.8 Mg C/ha, respectively). Although the cocoa and shade trees’ contributions were comparable, and together they contributed approximately 87.3–92.7 % of the total system’s biomass C, only the biomass C contribution from the shade trees correlated significantly with the system’s total biomass C (r = 0.9724, P < 0.001, Table 3). The lowest contribution to the system’s C storage was obtained from stumps in shaded systems in the Western region (Table 2).

**Table 2:** Mean C stocks ± standard error (Mg/ha) in cocoa trees, shade trees, stumps and litter components as influenced by region [Eastern (E), Western (W)] and system [shaded (S), unshaded (U)] and their interactions, (n = 12)

Factor	Treatment	Cocoa	Shade	Stumps	Litter
Region	E	15.2 ± 1.0	10.2 ± 6.4	0.16 ± 0.02	2.3 ± 0.2
	W	13.5 ± 1.0	16.4 ± 6.4	0.12 ± 0.02	2.4 ± 0.2
System	S	12.7 ± 1.1	13.3 ± 4.1	0.07 ± 0.02	2.6 ± 0.2
	U	16.1 ± 1.1	n.a.	0.21 ± 0.02	2.2 ± 0.2
Region * System	E * S	13.6 ± 1.5	10.2 ± 6.4	0.12 ± 0.03	2.3 ± 0.2
	E * U	16.9 ± 1.5	n.a.	0.19 ± 0.03	2.4 ± 0.2
	W * S	11.8 ± 1.6	16.4 ± 6.4	0.01 ± 0.03	2.9 ± 0.2
	W * U	15.2 ± 1.5	n.a.	0.24 ± 0.03	1.9 ± 0.2

Age of farms appearing as covariate in the statistical model used

Not applicable

**Table 3:** Pearson correlation coefficients (r) for linear relationships among biomass C components in cocoa ecosystems

	Ecosystem	Cocoa	Shade	Stumps
Cocoa	0.4936			
Shade	0.9724**	0.2801		
Stump	−0.1842	0.2948	−0.2536	
Litter	0.4945	0.5397	0.3703	−0.4871

Values with ‘**’ are significant at P < 0.01, and without symbol are not significant, (2—tailed test)

Overall, the mean carbon storage of cocoa trees was similar to that estimated for cocoa trees in a 30 year old cocoa system in Cameroon (14.4 Mg C/ha) reported by Norgrove and Hauser [27]. The present study estimated C stock in cocoa trees similar to those reported by Isaac et al. [30] as 10.3 Mg C/ha in an 8 year-old cocoa system in Ghana [30]. Isaac et al. [26] estimated the C storage of a 15 year-old cocoa system in Ghana as 16.8 and 15.9 Mg C/ha for a 25 year-old system, both of which agreed with the present finding that the average carbon storage of cocoa trees ranged between 11.8 and 16.9 Mg C/ha.

### Soil organic carbon stocks

Understanding the effects of land use/land cover changes on ecosystem functions is often inferred from changes in soil organic carbon. However, measurements of SOC have often been excluded in many studies on land-use change because of methodological uncertainties. Jones et al. [31] reported a measurement standard error of 1000 kg/ha for SOC, due largely to wide variation in the soil C estimation at deeper soil profiles. In the current study, uncertainty was reduced in the characterization of the soil C pools from the surface to 60 cm depth by measuring C stocks in different soil layers.

Table 4 presents the measured SOC contents for different layers to 60 cm depth. There were considerable variations in SOC contents between regions and systems. The soil organic C stocks ranges in the 0–20, 20–40 and 40–60 cm depths were 35.7–70.7, 15.0–46.7 and 11.5–31.3 Mg/ha, respectively. Clearly, the bulk of the SOC was concentrated in the topsoil, 0–20 cm depth. Moreover, SOC decreased with depth under all the factors. At all depths, soils in the W had the highest mean C stocks. Significantly (P < 0.05) higher SOC stocks were measured in E than W at all depths. The system of production affected SOC storage from the surface to 40 cm depth, but not between 40 and 60 cm (Table 4).

**Table 4:** Mean soil organic C stocks ± standard error (Mg/ha) at 0–20, 20–40 and 40–60 cm layers as influenced by region [Eastern (E), Western (W)], and system [shaded (S), unshaded (U)], (n = 12)

Factor	Treatment	0–20 cm	20–40 cm	40–60 cm
Region	E	40.2 ± 3.4	16.6 ± 3.6	14.3 ± 1.6
	W	58.4 ± 3.4	33.3 ± 3.6	25.7 ± 1.6
System	S	45.4 ± 3.4	19.0 ± 3.6	18.5 ± 1.6
	U	53.2 ± 3.4	30.9 ± 3.6	21.4 ± 1.6
Region * System	E * S	44.7 ± 4.8	18.2 ± 5.0	17.1 ± 2.2
	E * U	35.7 ± 4.8	15.0 ± 5.0	11.5 ± 2.2
	W * S	46.1 ± 5.1	19.9 ± 5.4	20.0 ± 2.4
	W * U	70.7 ± 4.8	46.7 ± 5.1	31.3 ± 2.2

Age of farms appearing as covariate in the statistical model used

### Total cocoa ecosystem carbon stocks and accumulation

Table 5 presents the mean C stocks distributed between the biomass and soil components of cocoa ecosystems. Total above-ground C stock in ecosystem biomass was estimated as the sum of the biomass C from cocoa trees, shade trees, stumps, and litter (Table 2). The total biomass C was highly variable in the cocoa ecosystems and ranged from a minimum mean value of 16.7 ± 2.2 Mg C/ha from unshaded cocoa systems in the Western region to a maximum mean value of 31.3 ± 2.2 Mg C/ha measured in shaded cocoa systems in the Eastern region (Table 5). Statistical analysis of the total system’s biomass C showed significantly higher C stocks in E than W and in shaded than unshaded systems (Table 5).

**Table 5:** Mean cocoa ecosystem carbon stocks ± standard error, distributed between the biomass and soil (0–60 cm depth) components according to region [Eastern (E), Western (W)], and system [shaded (S), unshaded (U)], (n = 12)

Factor	Treatment	Biomass C (Mg/ha)	Soil C (Mg/ha)	Total C (Mg/ha)
Region	E	25.2 ± 1.6	70.5 ± 5.4	95.7 ± 8.6
	W	18.4 ± 1.6	113.0 ± 5.4	137.7 ± 8.6
System	S	25.8 ± 1.6	83.7 ± 5.5	115.5 ± 8.6
	U	17.8 ± 1.6	99.8 ± 5.5	117.9 ± 8.6
Region * System	E * S	31.3 ± 2.2	79.3 ± 7.7	109.5 ± 12.0
	E * U	19.0 ± 2.2	61.7 ± 7.7	81.8 ± 12.0
	W * S	20.2 ± 2.3	88.1 ± 8.2	121.5 ± 12.0
	W * U	16.7 ± 2.2	137.8 ± 7.7	153.9 ± 12.1

Age of farms appearing as covariate in the statistical model used

Total SOC pools from the topsoil to 60 cm depth varied considerably from a minimum of 61.7 ± 7.7 Mg C/ha in unshaded cocoa system in the Eastern region to a maximum C stock of 137.8 Mg/ha in unshaded system in the Western region (Table 5). Results from this study estimated higher SOC stocks than the mean SOC value of 60.4 Mg/ha in Dawoe [23] for 0–60 cm depth of cocoa soils in the Ashanti region, Ghana. Cumulative (0–60 cm depth) SOC indicated significant (P < 0.05) variations between regions and also between management systems (Table 5).

The total ecosystem C stock of cocoa systems was estimated as the sum of soil C within 0–60 cm depth and above-ground biomass C (trees, stump and litter C). Total ecosystem C was higher in the Western region (137.7 ± 8.6 Mg C/ha) than in the Eastern region (95.7 ± 8.6 Mg C/ha). These C estimates are very high when compared with data from Dawoe [23]. This is attributed to the low soil C stocks (35.5–80.4 Mg C/ha) from 0–60 cm depth reported by Dawoe [23], that were equivalent to the estimated C stocks in the current study’s topsoil, 0–20 cm (35.7–70.7 Mg C/ha) (see Table 4). Notably, in the current study, the soils contributed between 3 and 5 times more C than the above-ground pools of the cocoa ecosystems. Given the age range (7–28 years) of farms used in the current studies, as well as the extensive cultivation of 1.45 million hectares of cocoa in Ghana [1], it appears that approximately 118.6–223.2 Gg C could be stored in cocoa systems with stands aged within 30 years, irrespective of the shade-management system.

**Fig. 2:**
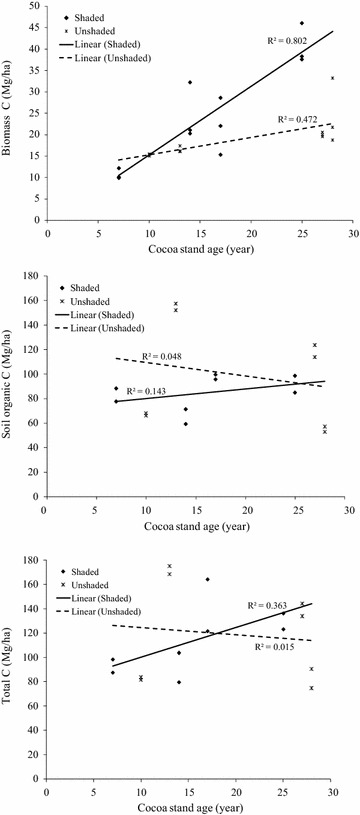
The C stocks dynamics in cocoa ecosystems (*lines*) with time as affected by cocoa systems (*scatter*) in the Eastern and Western regions of Ghana

The relative contribution of the cocoa systems (scatter) to the overall C stocks (line) in each component varied considerably when expressed on the basis of cocoa stand age (Fig. 2). The shaded and unshaded cocoa systems appeared to contain the same biomass stocks at stand age of 10 years in age. In the above-ground biomass C stocks, both shaded and unshaded cocoa systems increase with stand age but the contribution from the shaded systems to the overall biomass C trend was much higher than the unshaded system for cocoa stands older than 10 years (Fig. 2).

With respect to the effects of cocoa systems on soil C, there appears to be a general decline of the soil C stocks as time progressed. Whereas the shaded systems indicate a slight increase, the unshaded systems showed a slight decrease in soil C (Fig. 2). The two systems have similar soil C stocks at stand age of 25 years onwards.

The primary source of soil C is from litter and so the quantity and quality of the litter inputs affect the soil C dynamics [32]. Of the systems’ contribution to the total C in the ecosystems, the trend follows that of the soil C since the bulk of C (>80 %) is stored in the soil (Table 5). The trends indicate that total carbon in shaded and unshaded systems are the same at age 17 years, but the shaded system thereafter, increased in the total C higher than that of the unshaded system (Fig. 2).

## Conclusions

The need to quantify the carbon stocks in cocoa systems in Ghana is necessitated by the recent inclusion of the sector as an option that could result in a net increase in terrestrial carbon stocks. Hence, this paper estimated the carbon stocks in shaded and unshaded cocoa systems at different age categories; the fields were selected from the Eastern region (E) and Western region (W) of Ghana. Total ecosystem carbon was higher in the W than E. While the biomass C stock from shaded systems was twice that in unshaded systems, the two systems did not differ significantly with respect to total ecosystem C stocks. The bulk of the C stock was in the soil. The estimated high C stocks suggest that the cocoa sector holds a large amount of carbon and should be included in the national carbon accounting emission budget of Ghana.

## Methods

### Physiology of the study area

**Fig. 3:**
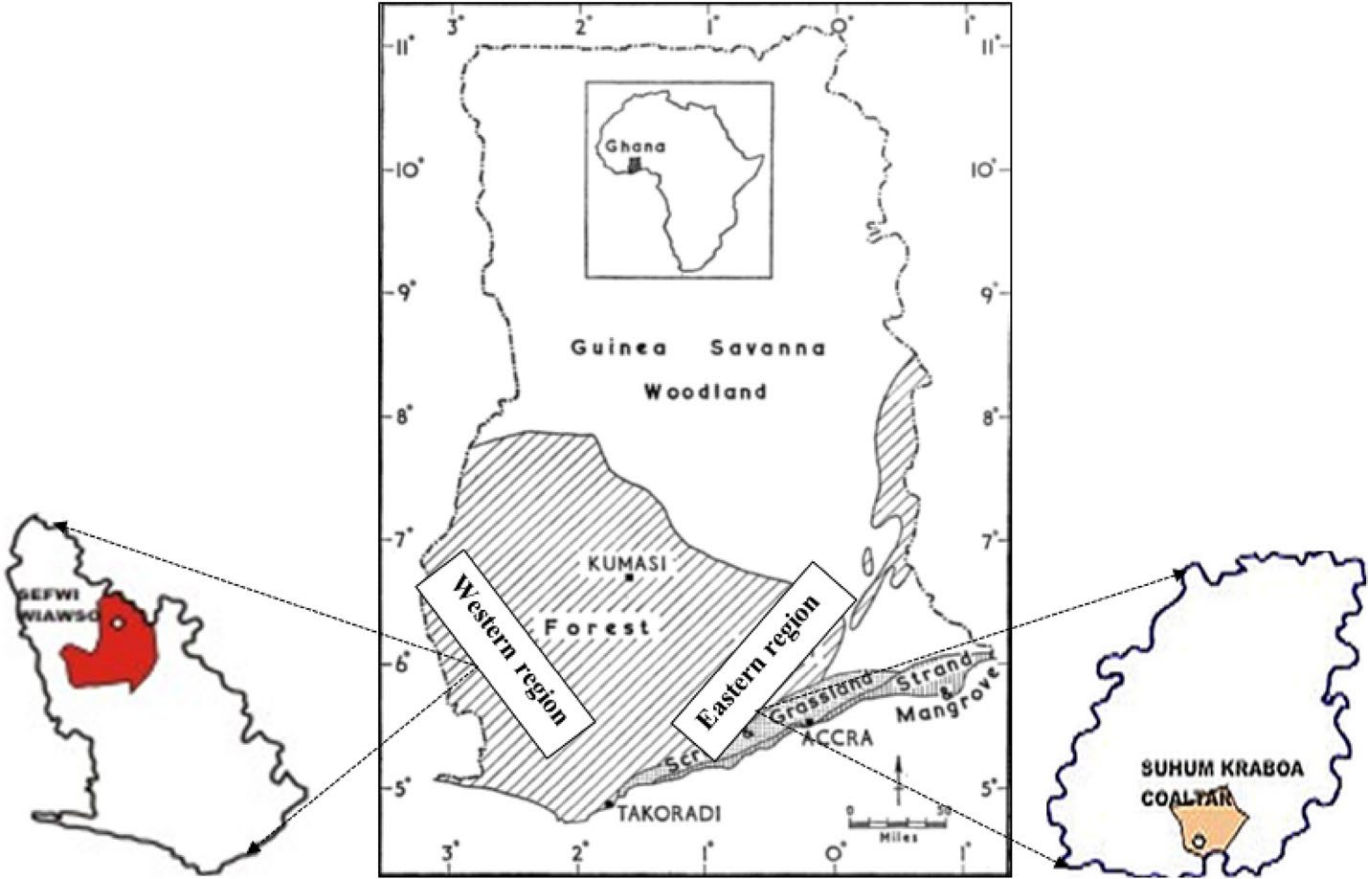
The position of Suhum and Sefwi-Wiawso where the cocoa farms were selected for the study: vegetation zones are based upon Taylor [33]. The vegetation zones of the Gold Coast, Accra

The field studies were carried out between July and October, 2011 in two regions of Ghana; the Eastern region at Duodukrom community in the Suhum district (6°2′N, 0°27′W), and the Western region at Anyinabrim in the Sefwi-Wiawso district (6°57′N, 2°35′W). Figure 3 presents the map of Ghana showing the regions and districts where the field studies were conducted.

The Eastern region covers a land area of 19,323 km^2^ representing 8.1 % of the total land area of Ghana. It is located between latitude 6° and 7°N and longitude 1°30′W and 0°30′E. The region lies within the wet semi-equatorial zone which is characterized by double-maxima rainfall in June and October. The natural vegetation of the region is humid deciduous forest. Temperatures in the region are high and range between 26 °C in August and 30 °C in March. The relative humidity which is high throughout the year varies between 70 and 80 %.

The Western region occupies a land area of 23,921 km^2^ which is approximately 10 % of the total land area of Ghana. The region lies in the equatorial climatic zone that is characterized by a double maxima rainfall occurring in May–July and September/October. Its vegetation is that of humid deciduous forest. The region is the wettest part of Ghana with an average rainfall of 1600 mm per annum and harbours about 24 forest reserves that account for about 40 % of the forest reserves in Ghana. The climate creates much moisture culminating in high relative humidity, ranging from 70 to 90 % in most part of the region. Temperatures range between 22 °C at nightfall and 34 °C during the day.

Thus, the two regions experience similar climate and vegetation. The major soils found in both regions are mostly well drained *Ochrosols* or *Oxisols* suitable for the production of industrial crops such as cocoa, pineapple, pawpaw cola nut and oil palm. However, the Eastern region has been producing cocoa long before cultivations started in the Western region.

### Selection of farms

Eight farms, comprising four from the Duodukrom community in the Suhum district of the Eastern region, and four from the Anyinabrim community in the Sefwi-Wiawso district of Western region were selected for sampling cocoa stands on the basis of shade management (shaded, unshaded). Selected farms had cocoa stand ages of 10, 14, 25 and 28 years in the Eastern region (E) and 7, 13, 17 and 27 years in the Western region (W).

At each farm, plot sizes of 30 × 90 m were demarcated for sampling. Two 30-m transects dividing the plot into three of 30 × 30 m (~0.23 acre or 0.09 ha) sub-plots were demarcated to give three pseudo-replications of each farm. The common shade tree species identified on the cocoa farms included *Terminalia ivorensis, Terminalia superba, Entandrophragma cylindricum, Entandrophragma angolense, Newbouldia laevis, Persea americana, Celtis mildbraedii, Cola nitida, Carica papaya, Palmae* sp., *Spondia smombin, Ficus exasperate, Citrus sinensis* (L.) Osbeck, *Acacia mangium,* and other forest tree species. Avocado (*Persea americana*) was the dominant shade tree in cocoa farms found in the Western region whilst *Newbouldia laevis* was the dominant shade tree in the Eastern region’s cocoa farms.

All trees were counted, and their diameters at breast height (DBH) measured, sorted and grouped into three diameter class sizes (upper, middle, and lower) relative to the DBH range of cocoa trees on the farms; 16 cocoa trees, comprising two cocoa trees per farm were randomly selected such that the diameter of one tree lay within the upper class and the other in the lower class for destructive sampling. The felled trees were each separated into trunks, branches and foliage (leaves, fruits); these parts were cut to smaller pieces, weighed in batches and then summed to give total component weight. Fresh leaf samples of the dominant shade trees found in each region were also taken. Based on the measured DBH and the biomass per tree of the 16 cocoa trees that were destructively sampled across all the study sites, an allometric relation was developed using regression techniques to estimate standing cocoa tree biomass. The general equation from FAO [34], recommended by UNFCCC [35], was used to estimate the above-ground biomass of the shade tree species.

1 $$AgB = \exp \left[ { - 2.134 + 2.530\ln \left( {DBH} \right)} \right]$$

where *AgB* denotes above-ground biomass, kg tree^−1^, and *DBH* = diameter at breast height, cm.

### Soil moisture and bulk density

Soil samples at 0–20, 20–40 and 40–60 cm depths were taken from a total of 16 plots comprising 2 micro-plots of (50 × 50 cm) that were established at random within the eight cocoa farms. Two core soil samples per depth were taken randomly at each micro plot using an auger after removing visible litter from the soil surface. Soil bulk density gives an indication of the level of soil compaction [36]. Soil bulk density and moisture contents at each sampling depth were determined on the undisturbed core samples, as outlined in Blake and Hartge [37].

### Texture

Another set of soil samples from the same micro-plots was air-dried for 72 h, and ground to pass through a 2-mm mesh sieve to yield the fine earth fraction for chemical analysis. The soil particle size distribution was determined by laser granulometry, using a Coulter LS230 particle size analyser connected to a Windows-based computer [38–40].

### Carbon concentration

Weights between 0.9–1.1, and 8.0–12.0 mg were taken respectively, from plant and soil samples the determination of C concentrations. The organic C concentration in the samples was determined using the Europa Roboprep connected to a VG 622 Mass Spectrophotometer.

### Carbon stocks

There are various C pools, or compartments, within cocoa ecosystems. These include the soil C pool, the litter C pool and the woody biomass C pool in trees. The quantity of C stored in each pool is reported as the C stock, and the sum of the C stocks from the different pools constitutes the total ecosystems C stocks. On each farm, the total biomass-C stock was estimated as the sum of the C stocks in cocoa tree components (root, stem, branch, and leaf litter), floor litter and shade trees (if any) as expressed in Eq. (2). The cocoa tree component-C stocks were calculated as the product of the mean C concentration and the biomass per hectare [41]. The mean C concentration of leaves of shade trees was used as the average C concentration of the whole shade tree in estimating the C stock of the shade trees.

2 $$Total C_{biomass} = \{ [(\% C_{root} \times root_{biomass} ) + (\% C_{stem} \times stem_{biomass} ) + (\% C_{branch} \times branch_{biomass} ) + (\% C_{leaf} \times leaf_{biomass} )]_{cocoa} + (\% C_{litter} \times litter_{biomass} ) + (\% C_{shade} \times Shade_{biomass} )\}$$

Soil C stocks were also calculated using the formula:

3 $$SOC = \mathop \sum \nolimits \rho_{i} \times d_{i} \times \% C_{i}$$

where *SOC* denotes soil organic carbon stock (Mg/ha); *ρ* = soil bulk density (g/cm^3^); *i* = 0–20, 20–40, and 40–60 cm sampling depth; *d* = depth over which the sample was taken (cm); and  %*C* = soil carbon concentration (%). The total cocoa ecosystem carbon stock for each farm/system was then estimated as the sum of Eqs. (2) and (3).

### Data analyses

The data were tested for normality using q–q plot with Anderson–Darling P values in MINITAB v16. Where the tested component C was found to be non-normal, the appropriate transformation was determined with the help of Box-Cox transformation and optimal or rounded lambda that suggested one of the following transformational method as appropriate: square root, reciprocal square root, natural logarithm or inverse transformation method, according to the skewness of the data [42]. Specifically, litter, stumps and total ecosystem C data were normal (P > 0.05) without transformation; biomass and soil C were inversely transformed; cocoa tree C was transformed with square root and shade tree C was normalized using natural logarithms. The transformed data were analysed by the Linear MIXED Model of IBM SPSS statistics 20th edition to determine significant differences between Eastern and Western regions and between shaded and unshaded systems as well as the interactions on carbon stocks controlling for the ages (covariate) of cocoa farms. The means were then estimated by restricted maximum likelihood (REML) and back-transformed to maintain the original form of the measurement. Correlation analyses by Pearson’s rank matrix were also carried out to determine any relationships among some of the ecosystem variables.
